# Assessing the Quality and Readability of Online Patient Information for Common Proctological Conditions

**DOI:** 10.7759/cureus.85595

**Published:** 2025-06-09

**Authors:** Shoaib S Saeed, Kayden Chahal, Gillian Smith, Naadir Nazar

**Affiliations:** 1 General Surgery, Maidstone and Tunbridge Wells NHS Trust, Royal Tunbridge Wells, GBR

**Keywords:** anal fissure, anal fistula, haemorrhoids, proctology, readability analysis

## Abstract

Background

Haemorrhoids, anal fissures, and anal fistulae are common benign proctological conditions that heavily rely on self-management strategies to prevent morbidity. Online access to various treatment options has empowered patients in this regard. This study was conducted to assess the quality and readability of the available online information.

Methods

An online search using the Google search engine (Google, Inc., Mountain View, CA, USA) was carried out with the following terms: ‘Treatment of Haemorrhoids’, ‘Treatment of Anal Fissure’, and ‘Treatment of Anal Fistula’. For each search term, the first 25 webpages developed for patient education were included. Thus, a total of 75 webpages were analysed for their quality using the DISCERN instrument and the Journal of the American Medical Association (JAMA) benchmarks. Their readability was assessed using the Flesch Reading Ease Score (FRES), Flesch-Kincaid Grade Level (FKGL), and the Simple Measure of Gobbledygook (SMOG).

Results

Across the three search terms, the average overall DISCERN score was 2.4 ± 0.8 (out of five), suggesting moderate to low-quality information. Only 16 (21%) webpages fulfilled all four JAMA benchmark criteria for quality. An average FRES score of 57.6 ± 9.0 indicated that the text was fairly difficult to read. FKGL and SMOG index levels of 8.0 ± 1.6 and 10.7 ± 1.0, respectively, correspond to the reading age range of 13- to 16-year-olds.

Conclusion

Available online information for patients with common proctological complaints is of suboptimal quality and is fairly difficult to read. It is essential to ensure that the information available meets high-quality standards and is readable by patients.

## Introduction

Haemorrhoids, anal fissures, and fistulae are benign anorectal disorders that can cause significant distress to patients [1]. In the United Kingdom (UK), the one-year prevalence of haemorrhoids is estimated at 10% [2]. Whilst prevalence data on anal fissures are not available, lifetime incidence is calculated at 11% [3]. Anal fistulae are less common, with a prevalence rate of fewer than 5 per 10,000 population, according to a systematic review of population-based studies conducted in Europe [4]. Patients presenting with bleeding, pain, or pruritus in primary care settings are often diagnosed with haemorrhoids, anal fissures, or fistulae, and receive guidance on conservative management. When primary care services fail, patients seek secondary care, which may include surgery - and surgery is associated with a high morbidity risk.

Upon diagnosis, patients should be directed to appropriate information about their condition. Patient information leaflets or other available physical resources may help in educating patients. However, many patients may resort to online sources for additional information. The exhaustive wealth of publicly available online health information has tremendous potential to educate and improve the health of patients; however, not all online sources are accurate or have optimal readability for patients. In fact, inaccurate and unreadable resources may even be harmful to patients [5]. Health literacy encompasses the ability of patients to communicate their health needs, understand instructions provided by healthcare professionals, and understand written health information [6]. Poor health literacy is associated with unhealthy lifestyle behaviours and a greater need for emergency services [7].

In the UK, online information provided by the government is written for a target reading age of nine years old [8]. Just under half (43%) of adults in the UK have a literacy level below that of an average seven- to nine-year-old, and 78% of adults have a numeracy level below this [9]. In England, 42% of 16- to 65-year-olds are unable to understand everyday health information [7]. It is essential to ensure that high-quality health information is written at an appropriate level for most of the population to understand in order to reduce health inequalities.

For a patient to be able to access, understand, and interpret online information, they must have access to the internet and understand information in a digital format. Even in today’s world, 8% of individuals in the UK do not feel confident using the internet, and 6% of UK households do not have internet access [9]. Furthermore, when searching for online information through a search engine, 92% of users will only view the first page of results, with the first five results taking over 75% of the web traffic [10]. Additionally, users read only 20%-28% of the text on a webpage [8]. These online resources may be the only sources of information that patients access. Online resources may guide their decision-making and self-care habits, and influence their choice of treatment and ongoing management. We aimed to assess the quality of online resources for common proctological conditions (haemorrhoids, anal fissures, and anal fistulae) to better understand the impact they have on patients and the overall delivery of care.

This article was previously presented as a meeting abstract at the 2023 ACPGBI Annual Scientific Meeting, July 3-5, 2023, in Manchester.

## Materials and methods

Web-based data collection

Web searches and subsequent data analysis were carried out in January 2023 by one author (GS). Searches were conducted using the Google search engine (Google, Inc., Mountain View, CA, USA) for the following three search terms: (1) Treatment of Haemorrhoids, (2) Treatment of Anal Fissure, and (3) Treatment of Anal Fistula.

Terms were kept simple, avoiding medical jargon, to most accurately reflect the terms patients might use to seek information online. The first 25 relevant webpages for each condition were analysed. Website URLs and subsequent analyses were recorded and stored in an Excel spreadsheet (Microsoft® Corp., Redmond, WA, USA).

Before the actual search, agreed exclusion criteria included sponsored search results, medical journals and publications, paid-access searches, or results not aimed at patients. Examples of included results were patient portals, health information websites, and charity sites. Webpages out of scope beyond the current exclusions included blogs by individual patients and patient forums. Upon review of the post-search results, certain websites were excluded as they pertained to the treatment of non-human disease, were duplicates of the same resource, or required sign-in credentials.

Quality and readability analysis

Once deemed fit for analysis, the contents of the included webpages were assessed for readability and quality. As defined by the Centre for Health Information [11], written healthcare information must possess three key quality attributes: (1) information should be clearly communicated, (2) information should be evidence-based, and (3) information should involve patients in the development of the materials. Our study focused primarily on the first two points via the use of several scoring tools.

DISCERN scores

DISCERN is a tool developed via expert analysis of consumer health information [12]. It serves as a guide for the standard of information on treatment choices. Three categories form the index: reliability, treatment, and overall rating. These were assessed via a 15-point questionnaire, with a final question requiring an objective overall assessment between 1 and 5 points (Appendix 1). The maximum score possible is 75, with different numerical ranges corresponding to a final rating ranging from ‘good’ to ‘very poor’. The mean and standard deviation of the scores for all webpages were calculated for the 15 questions, and an overall rating for all three conditions was obtained.

Journal of the American Medical Association (JAMA) benchmarks

Silberg et al. (1997) [13] described efficient, straightforward, and accessible criteria for accountability within quality assessment. These criteria are known as the JAMA benchmarks. Websites may be rapidly discounted or commended based on four key tenets: authorship, attribution, disclosure, and currency (Appendix 2). The mean, standard deviation, and mode were calculated for the final JAMA benchmark score for all three conditions.

Readability scores

Readability is defined as 'the ease with which a reader can understand a written text.' It varies based on both content and presentation. The way readability is calculated for webpages differs from how it is calculated for print media. The UK government’s best practice documentation [8] recommends aiming for a reading age of nine years. This recommendation is based on the fact that, in the UK, 7.1 million adults read at or below a nine-year-old level [14]. While there are several ways to assess readability, we used the following three (Appendix 3): (1) Flesch Reading Ease Score (FRES), (2) Flesch-Kincaid Grade Level (FKGL), and (3) Simple Measure of Gobbledygook (SMOG).

Flesch Reading Ease Score (FRES)

This tool considers total words, sentences, and syllables. Scores range from 0 to 100, with higher scores indicating superior readability. Scoring categories vary from the United States (US) fifth grade (easiest) to postgraduate university level (hardest) [15].

Flesch-Kincaid Grade Level (FKGL)

This tool considers the same variables as FRES but presents scores in the US ‘grade’ format. Scores indicate the number of years of education required to understand the text. Lower scores indicate easier readability, and there is no upper limit [15].

Simple Measure of Gobbledygook (SMOG)

This tool calculates the number of polysyllabic words (more than three syllables) and sentences. Like FKGL, it estimates the years of education needed to understand the text [16].

Statistical analysis

The mean and standard deviation of readability scores were calculated for all three conditions. Statistical analysis was conducted using Microsoft Excel.

## Results

Searches yielded approximately 2,780,000 results for 'Treatment of Haemorrhoids,' 12,300,000 results for 'Treatment of Anal Fissure,' and 15,300,000 results for 'Treatment of Anal Fistula.' After applying exclusion criteria - which included sponsored content, advertisements, academic journals requiring payment, non-human studies, duplicate entries, and sites requiring sign-in - the first 25 webpages were evaluated for each search term.

Across the three search terms, the average overall DISCERN score was 2.4 ± 0.8, suggesting moderate to low-quality information. Only 16 out of 75 (21%) webpages fulfilled all four JAMA benchmark criteria for quality. An average FRES score of 57.6 ± 9.0 indicated that the text was fairly difficult to read. FKGL and SMOG index levels of 8.0 ± 1.6 and 10.7 ± 1.0, respectively, correspond to the reading age range of 13- to 16-year-olds.

Haemorrhoids

A total of 11 webpages were excluded. Data are displayed in Table 1. Webpages on haemorrhoids had a mean overall DISCERN score of 2.28 ± 0.94, indicating moderate to low quality. The total DISCERN mean was 34.72 ± 9.57. Lower scores were particularly noted in areas concerning shared decision-making and detailed explanations of treatment risks and benefits. Scores varied widely among webpages (range: 22-52), representing heterogeneity in quality. Only 24% (6/25) of webpages on haemorrhoids met all four JAMA benchmarks: authorship, attribution, disclosure, and currency - indicating a notable gap in transparency and credibility.

**Table 1:** Quality and Readability Scores for Haemorrhoids JAMA, Journal of the American Medical Association; FRES, Flesch Reading Ease Score; FKGL, Flesch-Kincaid Grade Level; SMOG, Simple Measure of Gobbledygook

Website	Total DISCERN (Q1-15)	Overall DISCERN (Q16)	JAMA Benchmark Score (/4)	FRES	FKGL	SMOG
NHS	26	1	2	80.4	4.1	7.7
Mayo Clinic	41	3	3	46.5	8.5	10.9
NHS Scotland	40	3	2	52.2	9.4	12
BUPA	41	3	4	71.2	6.9	9.8
NIDDK	28	2	2	49.5	9	11.3
Germoloids	28	2	0	45.5	11.3	13.9
Health Harvard	30	2	2	50.4	9.7	12.5
WebMD	52	4	4	59.5	7.5	10.5
Medical News Today	39	3	4	48	9.2	11.6
Health Online	40	3	3	52.6	8.3	10.7
Colorectal Centre	47	3	0	56	8.8	11.7
Hopkins Medicine	31	2	0	68.7	6	9.4
Health Direct	38	3	2	53.6	8.7	11.3
Every Day Health	51	4	4	51.5	9.2	11.9
HSE Ireland	28	2	2	74.7	4.9	8.4
GSTT	33	2	2	73.8	5.3	9.2
Better Health	26	1	2	57.3	7.3	10.1
Wikipedia	48	3	4	42.3	9.9	12.2
Nuffield Health	31	2	0	61.8	7.8	11
Paitent.info	48	3	4	64.7	7.1	10.2
King Edward VII	33	2	1	53.2	9.2	11.9
Medline Plus	23	1	0	47.4	8.4	10.8
Boots	22	1	3	56.4	7.7	10.5
Choc	22	1	0	41.3	9.8	11.8
Bladder and Bowel	22	1	0	58.2	8.4	11.2
Mean	34.72	2.28	2.00	56.67	8.10	10.90
Standard Deviation	9.57	0.94	1.53	10.50	1.69	1.34

FRES ranged from 41.3 to 80.4; FKGL, from 4.1 to 11.3; and SMOG, from 7.7 to 13.9. A mean FRES of 56.67 ± 10.50 suggests that the text is 'Fairly Difficult' to read for the average reader. The mean FKGL score of 8.10 ± 1.69 suggests that most content is written at an eighth-grade reading level. The mean SMOG index of 10.90 ± 1.34 indicates that a reader would need at least an 11th-grade education to comprehend the material. This complexity exceeds the level recommended for general audiences.

Anal fissures

A total of 12 webpages were excluded. Data are shown in Table 2. Webpages on anal fissures had a mean overall DISCERN score of 2.36 ± 0.86 - higher than that for haemorrhoids - still indicating moderate to low quality. The total DISCERN mean was 34.72 ± 7.95, with scores ranging from 21 to 47. Lower scores were noted in areas concerning treatment options and the balance of risks and benefits. Only 16% (4/25) of webpages on anal fissures met all four JAMA benchmarks.

**Table 2:** Quality and Readability Scores for Anal Fissures JAMA, Journal of the American Medical Association; FRES, Flesch Reading Ease Score; FKGL, Flesch-Kincaid Grade Level; SMOG, Simple Measure of Gobbledygook

Website	Total DISCERN (Q1-15)	Overall DISCERN (Q16)	JAMA Benchmark Score (/4)	FRES	FKGL	SMOG
NHS	34	2	2	56.6	9.2	11.6
Mayo Clinic	40	3	3	59.3	7.3	9.8
WebMD	47	4	4	65.5	6.7	9.3
Cleveland Clinic	43	3	2	55.6	7.8	10
BUPA	37	3	4	65.3	7.3	9.9
FASCRS	30	2	1	50.8	9.9	11.8
Better Health	34	2	2	58	7.2	8.9
Healthline	44	3	2	58.6	7.4	9.7
Hopkins Medicine	28	2	0	55.8	7.7	9.7
GOSH	24	1	1	60.4	8.7	10.8
Cambridge Bowel Clinic	26	1	0	65.4	7.8	9.9
Family Doctor	31	2	3	63.5	7	9.1
NI Direct	22	1	0	59.8	7.6	9.8
NHS Wales	42	3	2	56.1	9.3	11.4
Patient.info	44	3	4	64.2	7.4	9.8
Colorectal Centre	39	3	0	47.9	9.7	11.4
Cedars Sinai	35	3	1	70.4	5.6	8.4
Choc	23	1	0	59.8	7.1	9
Imperial NHS	43	3	1	70.9	5.2	8.4
ADA	41	3	4	52.7	8.9	10.7
UCSF health	21	1	0	64.7	6.7	9.7
Health Direct	30	2	2	70	5.8	8.7
MKUH	35	3	0	66.7	7.2	9.5
Very Well Health	45	3	2	64.1	6.8	9.5
My Health Alberta	30	2	2	68.9	6.3	8.7
Mean	34.72	2.36	1.68	61.24	7.50	9.82
Standard Deviation	7.95	0.86	1.41	6.20	1.20	0.98

FRES ranged from 32.7 to 71.3; FKGL, from 5.7 to 13.3; and SMOG, from 9.9 to 15.4. The mean FRES of 55.04 ± 8.93 classifies the text as 'Fairly Difficult,' suggesting that the material may be challenging for the average reader. The mean FKGL score of 8.62 ± 1.81 suggests that the content is written at the level of an eighth- to ninth-grade reader, which may not be accessible to all audiences, particularly those with lower literacy levels. The mean SMOG index of 11.56 ± 1.56 indicates that a reader would need at least 12 years of education to comprehend the material. This is, again, a complexity level too high for the general public.

Anal fistulae

A total of 12 webpages were excluded, as demonstrated in Table 3. Webpages on anal fistulae had a mean overall DISCERN score of 2.60 ± 0.82 - higher than for haemorrhoids and fissures - still indicating moderate to low quality. The mean total DISCERN score was 35.40 ± 7.00, with values ranging from 26 to 53. Only 24% (6/25) of webpages on anal fistulae met all four JAMA benchmarks, underscoring a recurring theme of poor adherence to ethical standards of transparency and credibility in these resources.

**Table 3:** Quality and Readability Scores for Anal Fistulae JAMA, Journal of the American Medical Association; FRES, Flesch Reading Ease Score; FKGL, Flesch-Kincaid Grade Level; SMOG, Simple Measure of Gobbledygook

Website	Total DISCERN (Q1-15)	Overall DISCERN (Q16)	JAMA Benchmark Score (/4)	FRES	FKGL	SMOG
NHS	40	3	1	56.7	8.6	11.9
Cleveland Clinic	39	3	2	57.2	8.1	11.1
Mayo Clinic	39	3	4	50.9	8.6	11.4
WebMD	32	2	4	71.3	5.7	9
BUPA	53	5	4	60.8	8.2	11.7
Hopkins Medicine	26	1	0	60.5	7.2	11.1
GSTT	36	3	0	70.1	6.1	9.8
Patient.info	40	3	4	55.8	8.7	11.9
NHS Wales	43	3	1	54.8	9.2	12.3
FASCRS	36	3	1	32.7	13.3	15.4
Cedars Sinai	30	2	1	64	6.8	9.9
Wikipedia	45	3	4	43.3	10.6	13.3
Crohn's Colitis Foundation	26	1	1	49.9	9.2	12.4
Guts Charity	39	3	0	53.5	10.2	12.8
Winchester Hospital	28	2	1	53.6	7.3	9.2
IFFGD	28	2	2	42	11.1	13.2
Health Direct	36	3	2	54.3	8.3	11.5
Samitivej Hospitals	41	3	2	45.6	11.4	13.9
Medical News Today	43	3	4	48.9	8.5	11
UHCW	35	3	1	65.8	5.9	9.3
Family Doctor	28	2	1	65.8	6.8	9.9
Top Doctors	31	2	2	60.8	7.6	10.4
Colorectal Centre	37	3	0	51.2	9.6	12.2
One Welbeck	27	2	0	54.8	9	12.1
Surgery UCSF	27	2	1	51.7	9.4	12.3
Mean	35.40	2.60	1.72	55.04	8.62	11.56
Standard Deviation	7.00	0.82	1.46	8.93	1.81	1.56

FRES ranged from 47.9 to 70.9; FKGL, from 5.2 to 9.9; and SMOG, from 8.4 to 11.8. The mean FRES of 61.24 ± 6.20 classifies the text as 'Standard,' suggesting that the material is fairly accessible but may still pose challenges for readers with lower literacy levels. The mean FKGL score of 7.50 ± 1.20 suggests that the content is written at a seventh- to eighth-grade level, which is slightly above the UK-recommended sixth-grade level for patient education materials. The mean SMOG index of 9.82 ± 0.98 indicates that a reader would need nearly 10 years of education to comprehend the material - once again pointing to a complexity level higher than ideal for the general public.

## Discussion

The goal of patient education is to empower individuals to manage the day-to-day realities of their illness and enhance their overall health and well-being [17]. In a digital age, and in the context of evolving digital literacy, patients increasingly rely on online information. High-quality, easy-to-understand information can reassure patients and facilitate proper self-care, thereby reducing morbidity. Additionally, this is a cost-effective, population-level intervention associated with a positive health economic impact [18]. However, the lack of regulation, peer review, and the heterogeneity of sources for published information call into question the quality and readability of online information.

Symptoms of benign anorectal diseases can vary in severity and complexity. As such, treatment options can range from conservative or medical management to complex surgical procedures. High-quality online resources present an opportunity to guide these treatment options and complement the advice received from consulting clinicians. Additionally, due to the potentially embarrassing nature of the disease and high rates of self-diagnosis, some patients may avoid seeking professional medical care and eventually present with more advanced symptoms [19,20]. We can infer that these individuals are likely to rely on online resources to help them understand and manage their symptoms.

While haemorrhoids are the most common of the three conditions, followed by fissures and then fistulae, the volume of webpages for each condition showed the opposite trend, with fistulae returning 15.3 million hits compared to 12.3 and 2.78 million for fissures and haemorrhoids, respectively. This possibly reflects the fact that a lack of widespread consensus in the management of fistulae, along with the complexity of the disease [1], has led to more extensive online research by patients. This raises the possibility that the majority of webpages target the professional community rather than being aimed at patient education.

This study showed that webpages for all three conditions scored similarly across all tools. The mean overall DISCERN score was identical for haemorrhoids and fissures (34.72), and slightly higher for fistulae (35.4). All these scores suggest moderate- to low-quality content. Only 21% of webpages were fully compliant with JAMA benchmarks. This small percentage, along with a general lack of overall adherence (mean: 1.8 ± 1.45), suggests insufficient information and unreliable sources with poor author attribution. This subverts the tenets of evidence-based practice. Because these resources are the most easily accessed online, patient self-mismanagement could pose a significant potential concern.

Similar to quality assessment, all readability scores were very similar for information pertaining to haemorrhoids, fissures, and fistulae. The mean reading age in the UK is that of a seven- to nine-year-old, and the ability to access and understand information in digital form is an additional required skill. The UK government targets online written information at a reading level of a nine-year-old [8]. However, our study shows that the most accessed webpages generally do not comply with this recommendation. Pooled FKGL readability scores indicated that online resources were targeted at a reading level of eighth grade (13- to 14-year-olds), whereas SMOG scores suggested that to understand the online resources, a reader would require over 10 years of formal education. If the reading level of the resources does not match that of the intended audience, there is a risk of poor comprehension or even misinterpretation of key information.

Readers' attention spans can be as short as eight seconds; additionally, only around a quarter of the information on a webpage is actually read in most cases [4,21]. Patients are likely to read only the headlines and key points, which is why it is important to use a tool that gives a clear indication of reading ease, such as the FRES. Information on haemorrhoids (FRES 56.67) and anal fistulae (FRES 55.04) was categorised as ‘fairly difficult to read’ or at the 10th-12th grade (14- to 18-year-old) level. Information on anal fissures (FRES 61.24) had a slightly higher readability and was classified as ‘easily understood’ or at the eighth to ninth grade (13- to 15-year-old) level. All analysed sources of information fell beyond the scope of the average reading age in the UK. This could potentially harm patients, widen health inequalities, and exacerbate morbidity associated with poor literacy.

We note some limitations in our study. While Google remains the most widely used online search engine, it is not the only source of online information for patient self-education. Information disseminated via other media formats, such as videos or podcasts, must also be evaluated for accuracy and readability. There is documented criticism of DISCERN, the JAMA benchmarks, and readability scores. DISCERN scores may not correlate well with scientific accuracy, and these scores do not consider visual or numerical data [12]. The JAMA benchmarks may be too simplistic [22]. Readability scores reflect simplicity, but not whether the text is well written or engaging [23]. We only had one data collector, which limited inter-observer bias; however, we recognise the possibility of intra-observer bias and attempted to rigorously follow a consistent approach to mitigate this. Finally, quality and readability are only relevant if patients consume content that pertains to a disease they actually have. It is of paramount importance that patients engage with resources after receiving a diagnosis from a trained professional, informed by clinical history and examination.

This study raises several pertinent points regarding health promotion policies in the field of proctology. Ideally, online information should be evidence-based and peer-reviewed, while remaining accessible. Government and regulatory bodies should work in tandem to set and maintain quality and readability standards. Moreover, artificial intelligence is evolving rapidly and has the potential to revolutionise patient health literacy [24]. Indeed, at the time of writing this article, the first hit on Google was an artificial intelligence-generated overview of the respective conditions - which was not the case at the time of data collection.

## Conclusions

Online information freely accessible to patients for the self-education and self-management of haemorrhoids, anal fissures, and fistulae is generally of low quality and not easily readable. This poses a risk of worsening health inequalities and compromising patient care. We highlight the importance of regulated, high-quality health information as an intervention to improve outcomes in benign anorectal diseases.

## Figures and Tables

**Figure 1:**
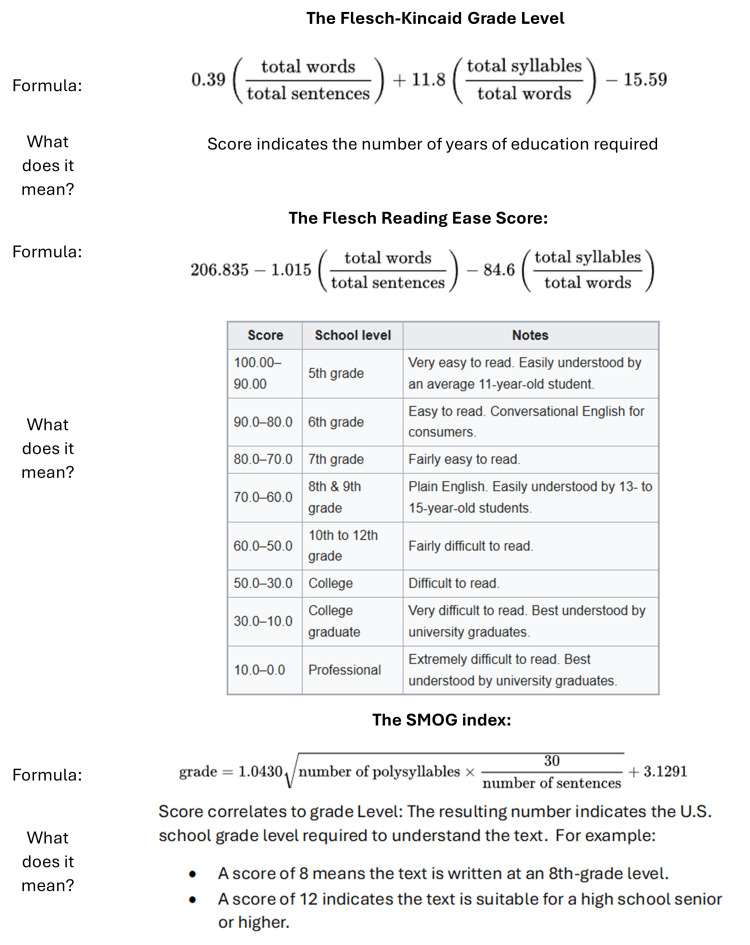
Readability scores Figure credit: [15,16]

## Appendices

Appendix 1

**Table 4:** The DISCERN tool Table credit: [12]

All questions rated using the following scale: 1 = no, 3 = partially, 5 = yes	
Section	Question (/5)
Section 1: Is the publication reliable	1. Are the Aims clear?
	2. Does it achieve its aims?
	3. Is it relevant?
	4. Is it clear what sources of information were used to compile the publication (other than the author or procedure)?
	5. Is it clear when the information used or reported in the publication was produced?
	6. Is it balanced or unbiased?
	7. Does it provide details of additional sources of support and information?
	8. Does it refer to any area of uncertainty?
Section 2: How good is the quality of information on treatment choices?	9. Does it describe how each treatment works?
	10. Does it describe the benefits of each treatment?
	11. Does it describe the risks of each treatment?
	12. Does it describe what would happen if no treatment were used?
	13. Does it describe how the treatment choices affect the overall quality of life?
	14. Is it clear that there may be more than one possible treatment choice?
	15. Does it provide support for shared decision-making?
Section 3: Overall rating of the publication	16. Based on the answers to all above questions, rate the overall quality of the publication as a source of information about treatment choices.

Appendix 2

**Table 5:** The JAMA Benchmarks Table credit: [25]

Criteria	Description
Authorship	Authors and contributors, their affiliations, and relevant credentials should be provided
Attribution	References and sources for all content should be listed clearly, and all relevant copyright information noted
Disclosure	Website 'ownership' should be prominently and fully disclosed, as should any sponsorship, advertising, underwriting, commercial funding
Currency	Dates that the content was posted and updated should be indicated

Appendix 3
